# Supplementary material for: Ending violence against women: Help-seeking behaviour of women exposed to intimate partner violence in sub-Saharan Africa
Source: PLoS One. 2023 Oct 5;18(10):e0291913. doi: 10.1371/journal.pone.0291913 (PMC10553277; doi:10.1371/journal.pone.0291913)
Supplement: Attachment — Submitted filename: Response to Reviewers Comments_R1.docx [file pone.0291913.s001.docx]

**RESPONSE TO REVIEWERS COMMENTS**

Reviewer #1: The paper is an interesting read and quite well written. However, there are few points that authors might want to consider:

1. Lines 137-138 mention about interventions that were introduced in Sub Saharan Africa to deal with the problem of intimate partner violence. Authors should elaborate on the nature and enforcements of these interventions.

Response: Thank you. We have addressed this comment.

2. Lines 139-140 highlights that the key intervention is the support system for women facing IPV. Authors need to explain whether this support system is formal or informal? Moreover, if there exists a formal support system, discuss its implementation and effectiveness.

Response: Thank you. The kind of support systems has been indicated, as well as its implementation and effectiveness.

3. Some information regarding the proportion of females experiencing IPV in each country must be presented and its connection with the proportion of women seeking out support should be discussed.

Response: Thank you. We have addressed this comment.

4. According to Figure 1, 35.89% of women experiencing IPV in SSA sought for help. Table 2 reveals that approximately one third of the women corresponding to almost all sub categories sought help after experiencing IPV. So pooling of cross sectional data across SSA reflects a monotonous sort of finding. The analysis can be made interesting by adding country specific details especially of those with higher/lower prevalence than average values. Pooling cross sections must present some details that are normally missed out in country specific analysis.

Response: Thank you. We have provided supplementary containing results for the predictors of help seeking for IPV among women in Mali and Tanzania (Table S1).

5. Mali and Tanzania experience lowest and highest prevalence of help seeking. Relate these findings to the specific support mechanisms available for females.

Response: Thank you. We have addressed this comment.
